# Accumulation of Atmospheric PAHs in White Mustard – Can the Seeds Be Affected?

**DOI:** 10.1007/s00128-024-03895-w

**Published:** 2024-05-11

**Authors:** Katalin Hubai, Nora Kováts, Bettina Eck-Varanka, Selenge Tumurbaatar, Gábor Teke

**Affiliations:** 1 Centre for Natural Sciences, University of Pannonia, Egyetem Str. 10, 8200 Veszprém, Hungary; 2 ELGOSCAR-2000 Environmental Technology and Water Management Ltd., 8184 Balatonfuzfo, Hungary

**Keywords:** Air pollution, Diesel exhaust, Polycyclic aromatic hydrocarbons, Bioaccumulation, *Sinapis alba*, Bioconcentration factor

## Abstract

Traffic-related particulate matter emissions have been considerably reduced due to stringent regulations in Europe. However, emission of diesel-powered vehicles still poses a significant environmental threat, affecting rural ecosystems and agriculture. Several studies have reported that polycyclic aromatic hydrocarbons (PAHs), a group of potentially toxic organic compounds, can accumulate in crops and vegetables. In our study, white mustard (*Sinapis alba* L.) plants were experimentally treated with an extract of diesel exhaust. PAH concentrations were measured in the different plant compartments (stems, leaves and seeds), bioconcentration factors (BCFs) were also calculated. Significant accumulation was measured in the leaves and seeds, stems showed lower accumulation potential. All plant matrices showed high tendency to accumulate higher molecular weight PAHs, BCF was the highest in the 6-ring group. The fact that considerable accumulation was experienced in the seeds might show the risk of cultivating crops nearby roads highly impacted by traffic-related emissions.

PM mass concentrations have shown a diminishing tendency in Europe due to more stringent regulations coupled with technological improvements (Daellenbach et al. 2020), however, pollution from traffic, especially emission of diesel-powered vehicles, poses a serious environmental concern in Europe (Matthias et al. 2020). Particles emitted by incomplete combustion carry a wide range of potentially toxic organic compounds including polycyclic aromatic hydrocarbons (PAHs) (Caumo et al. 2022). Diesel engine exhaust was classified as carcinogenic to humans by the International Agency for Research on Cancer (IARC 2014). Traffic-related PAH emissions have been reportedly affecting rural environments, including agricultural fields (Dan-Badjo et al. 2007).

A wide range of studies have assessed the magnitude and pattern of atmospheric PAH bioaccumulation in vegetables (e.g. Ashraf and Salam 2012; Jia et al. 2018). These studies are vital as dietary uptake is considered the major exposure pathway of PAHs in comparison to inhalation (Bansal and Kim 2015). Tesi et al. (2021) for example reported that vegetables consumed in some parts of Nigeria could be unsuitable for consumption based on their PAH, especially benzo(a)pyrene content.

It is a very important question how accumulated PAHs are transferred within the plant’s body. Most studies assess accumulation in contaminated soils via the soil-root-stem system. Wu et al. (2019) measured PAHs concentrations in grains and other parts of winter wheat cultivated in soils artificially spiked with phenanthrene, anthracene and pyrene. Considerable accumulation was observed in the grains, depending on the plant growth stage.

PAHs from the atmosphere can be actually transported to the soil, providing an additional exposure route for plants (Kulhánek et al. 2005). Al-Nasir et al. (2022) assessed PAH contamination sources in soil and vegetables and found traffic emission as one of the main contributors. Nevertheless, direct uptake of PAHs from the atmosphere represents the main exposure pathway (Desalme et al. 2011).

Studies reporting the partition of accumulated PAHs in different compartments of higher plants after direct exposure to atmospheric PAHs are rather limited. As such, the main question of the study was to determine if atmospheric PAH intake can influence the quality and safety of seeds in crop species. Of Brassicacea species, white mustard (*Sinapis alba* L.) was selected for the test. It is not only a widely distributed crop species, but also, it is often grown in traffic-impacted areas, in the vicinity of main roads. It has a relatively short life cycle. Different Brassicaceae species have been widely studied and generally proved good accumulators of PAHs (Xiong et al. 2017; Zhang et al. 2018).

## Materials and Methods

PM2.5–10 sample was collected using a 20-year-old diesel-powered, light-duty vehicle equipped with diesel particulate filter (environmental standard EURO3). The vehicle was operated at idling for 6 × 10 min, emitted particles were collected about 1 m from the tailpipe applying a KÁLMÁN PM_2.5_ sampler (flow rate 32 m^3^/h). Six consecutive samples were collected on quartz filters (Whatman QMA, diameter 150 mm). The 6 filters were used as a composite sample. Aqueous extract was prepared as follows: filters were cut into small pieces and placed in beaker with 1000 mL high purity water. Extraction took 24 h at room temperature, during that time the beaker was stirred several times. Finally, the extract was filtered through 0.45 µm pore size filter (GN-6 Membrane, 0.45 µm Hydrophilic mixed cellulose esters) and stored at − 20°C until use.

Test plants were cultivated and treated according to the protocol specified by the No. 227 OECD GUIDELINE FOR THE TESTING OF CHEMICALS: Terrestrial Plant Test: Vegetative Vigour Test. The Guideline has been adopted to test the potential phytotoxic effects of atmospheric PM and to assess bioaccumulation of atmospheric PM-bound PAHs (Teke et al. 2020). The Guideline enlists white mustard among the recommended test species (Annex 2). Mustard seeds were procured from Szentesimag Co. (from ecological farming).

Shortly, test plants were cultivated in 15 cm containers (10 containers were used, with 3 plants per each pot). During cultivation and treatment, test plants were kept in a glasshouse, environmental conditions were in concordance with the prescriptions of the Guideline (temperature: 22 °C ± 10°C; humidity: 70% ± 25%; photoperiod: minimum 16 h light; light intensity: 350 ± 50 µE/m^2/^s).

Plants were treated with the PM extract by spraying it on the above-ground parts. For spraying, a CONXIN Q1P-CX01-380 portable electric paint spray gun was used, with application volume of 5 mL/pot/treatment. During spraying, soil of each pot was covered to avoid contamination. First treatment was applied when plants reached the 4 true leaf stage, followed by consecutive treatments every week (as such, sequence of treatments was: Day0, Day7, Day14, Day21, Day28, Day35, Day42, Day49, Day56, Day63, Day70, Day77, Day84, Day91 and finally, Day 98). Last treatment was applied at the time of ripening of the seeds. Plant samples were immediately taken to the laboratory, washed with ionic load-free water and frozen (− 20°C) until analysis.

MSZ 1484-6:2003 standard (MSZ 1484-6:2003: Testing of waters. Determination of polycyclic aromatic hydrocarbons (PAH) content by gas chromatographic-mass spectrometry, was followed to measure the PAH concentrations in aerosol extract. The analysis of accumulated PAHs in plant samples was carried out based on MSZ EN 15527:2009 (Characterization of waste. Determination of polycyclic aromatic hydrocarbons (PAH) in waste using gas chromatography mass spectrometry (GC/MS). In our measurements an HP-6890 gas chromatograph was coupled to an HP-5973(Agilent Technologies, Palo-Alto, USA) quadrupole mass spectrometer (low-resolution single MS).

In order to prepare the plant samples, a composite sample was made from each plant part (leaves, stems and seeds), using the 30 test plants. Plant material was homogenized, and 10 g was grinded with 10 g anhydrous sodium sulphate in a ceramic mortar and was extracted with 20 mL *n*-hexane three times for 20 min with Ultra-sonic extraction. As the next step, 10 mL acetone was added to the samples and were spiked with 100 μl of 0.01 μg/mL deuterated PAH surrogate mixture (Naphtalene-d8, Acenaphthene-d10,Phenanthrene-d10, Chrysene-d12, Benzo(a)pyrene-d12, Perylene-d12; Restek Corporation, USA). After the extraction the sample extract was concentrated in a dry nitrogen stream to 1 mL. With each sample an additional solid phase silica gel and alumina oxide sample clean-up was performed.

Quality control: Measurements were designed in harmony with the laboratory accreditation to ISO/IEC 17025:2018, and with the laboratory’s internal quality management system guidelines. During the analysis of the samples the following parameters were checked: Selectivity, Precision (Repeatability), Accuracy, Linearity, Limit of detection (LOD). Limit of quantifications (LOQ).

The LOD values of accumulated PAHs in plant samples were between 0.01 μg/kg to 0.015 μg/kg and in aerosol extract between 0.00015 to 0.0003 μg/L. The LOQ values in plant samples were ranged from 0,031 to 0.05 μg/kg and 0.00029 to 0.001 μg/L in aerosol filter water extract samples.

Pearson method was used to calculate the correlation between the accumulated PAH amounts in different plant parts and the PAH concentration of the extract.

Based on analytical results, Bioconcentration Factors (BCF) were calculated comparing PAH concentrations in the seed/extract, leaf/extract and stem/extract. The following equation was used (Kacálková and Tlustoš 2011):

$$BCF=\frac{\mathrm{PAH\, concentration\, in\, plant\, sample}\,[\mathrm{\mu g}/{\text{kg}}]}{\mathrm{PAH
\, concentration\, in\, the\, PM\, extract}\,[\mathrm{\mu g}/{\text{L}}]}$$

## Results and Discussion

Measured PAHs concentrations are given in Table 1. Altogether 19 PAHs were detected in the extract as well as in the plant matrices including the 16 priority PAHs enlisted by US EPA.

Phenanthrene, fluoranthene and pyrene were found in relatively high concentrations (phenanthrene 39.9 µg/kg in seeds, 39 µg/kg in leaves and 18.2 µg/kg in stems; fluoranthene 28.8 µg/kg in seeds, 25.7 µg/kg in leaves and 11.8 µg/kg in stems, finally pyrene 25.7 µg/kg in seeds, 16.7 µg/kg in leaves and 6.7 µg/kg in stems). Although naphthalene is generally reported as one of the dominant PAHs in bioaccumulation studies (Busso et al. 2018), it was found in low concentrations in all three parts evaluated.

Fellet et al. (2016) analysed leaves of six evergreen ornamental shrubs collected in Italy during winter and found the dominance of naphthalene, phenanthrene, fluoranthene, fluorene and pyrene in high traffic areas. Similarly, Ratola et al. (2009) reported the dominance of PHE, FLT, NAP and PYR in Pinus samples collected from Portugal and Spain. Atmospheric accumulation of three-ring PAHs (acenaphthene, acenaphthylene, fluorene, phenanthrene and anthracene) in winter wheat grains was reported by Tian et al. (2018).

Comparing the accumulation of PAHs in the different above-ground parts, concentration of each individual PAH was lower in the stem than in the leaves (Table 1), also, accumulation pattern was somewhat different. There was significant correlation between the concentration of accumulated PAHs in seeds and leaves (t = 3.4539, df = 3, *p* = 0.041, R^2^ = 0.8939). However, non-significant correlations were calculated between seed and steam concentrations (t = 2.2871, df = 3, *p* = 0.1062, R^2^ = 0.7972) as well as between leaf and steam concentrations (t = 1.7116, df = 3, *p* = 0.1855, R^2^ = 0.7029).

Wei et al. (2021) examined the accumulation capacity of 11 crop species, including Brassicaceae family (pakchoi: *Brassica campestris* L. ssp. chinensis Makino and broccoli: *Brassica oleracea* L. var. botrytis L.). In general, higher PAH concentrations were determined in leaves than in other tissues for most of the investigated plants. High-molecular-weight PAHs seemed to be more readily absorbed from the atmosphere by leaves than other parts. Uptake will strongly depend on the morphological traits of leaves (reviewed by Tian et al. 2019) such as shape, epicuticular wax content and surface roughness. White mustard has elongated and slightly rough leaves which most possibly will favour PAH accumulation.

It has been reported that a relative balance exists between PAHs in leaves and PAHs in the atmosphere (Barber et al. 2004). PAHs are volatile and evaporate from the leaves because of the high temperatures in spring and summer, whereas notable condensation phenomena are caused by low temperatures in fall and winter (Nakajima et al. 1995). The stomata of the leaves are critical organs for the exchange between plant leaves and the external environment, which directly affects the content and distribution characteristics of the leaves. Our sampling time was summer, and the temperature was high and the stomata were fully open. As such, volatilization of PAHs from the leaves could be postulated.

Li et al. (2009) cultivated rice plants in benzo(a)pyrene spiked soil in an air-quality controlled glasshouse and also placed pots being exposed to polluted urban air. Accumulation was assessed both in the grains and in the stem/leaf compartment. While no accumulation was found in rice grains cultivated in the glasshouse, outdoor pots showed detectable uptake. However, concentration of accumulated PAHs was about 2 magnitude higher in the stem/leaf compartment than in the grains (185.0 vs. 1.5 µg/kg). Wang et al. (2020) proved the transport of PAHs from vegetative tissues (stem and leaves) to winter wheat grains.

**Table 1:**
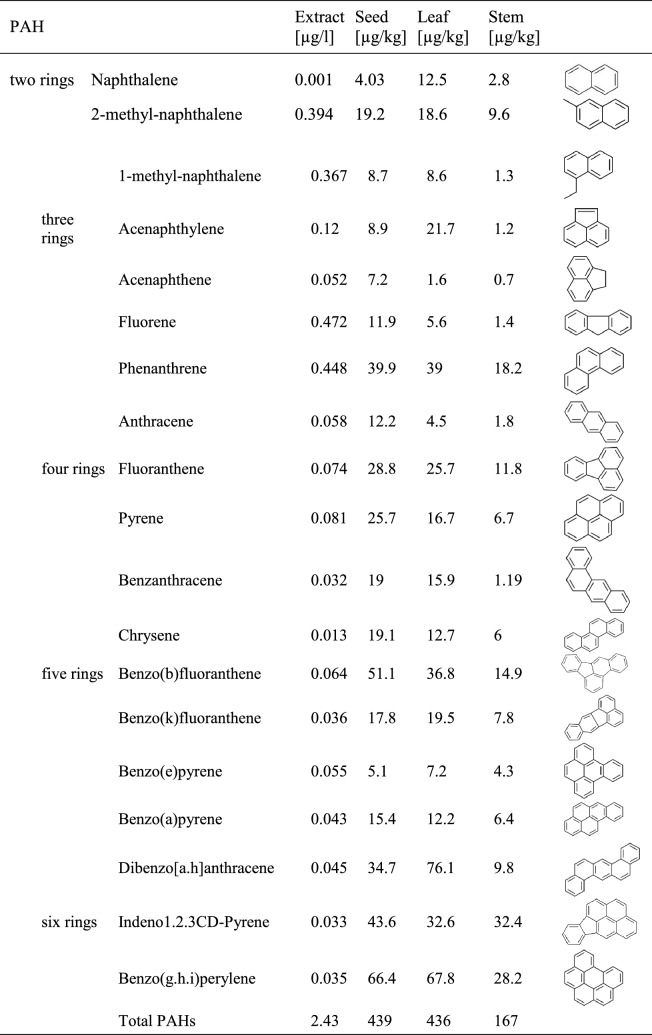
Concentration of PAHs in the aerosol extract and in the plant samples

**Fig. 1:**
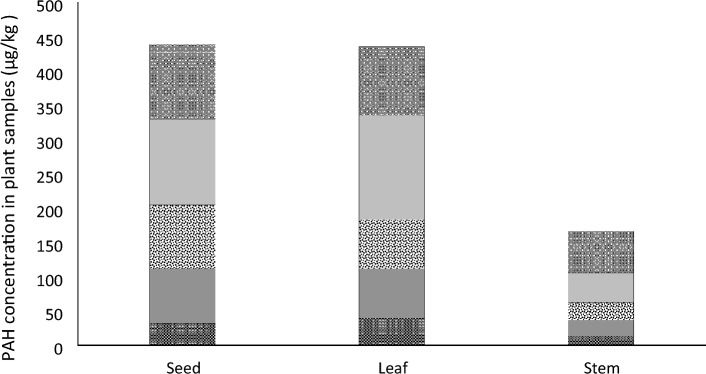
Concentration of different molecular weight PAHs groups in the tested plant matrices

Concentration of different molecular weight PAHs groups is illustrated in Fig. 1. A wide range of studies detected lower molecular weight compounds in different plant materials exposed to air pollution. Zhang et al. (2022) report the prevalence of 2- to 3-ring PAHs in crops of peri-urban farmlands, also showing the contribution of vehicular emission. In our study, in contrary to most of the reported results, HMW PAHs (5- and 6-ring compounds) dominated all three samples.

Of 5-ring PAHs, benzo(b)fluoranthene appeared in the highest concentrations (51.1 µg/kg in seeds, 36.8 µg/kg in leaves and 14.9 µg/kg in stems). It was followed by dibenzo(a.h)anthracene (34.7 µg/kg in seeds, 76.1 µg/kg in leaves and 9.8 µg/kg in stems). Of 6-ring PAHs, indeno1.2.3CD-pyrene and benzo(g.h.i)perylene were detected, both occurring in high concentrations (indeno1.2.3CD-pyrene: 43.6 µg/kg in seeds, 32.6 µg/kg in leaves and 32.4 µg/kg in stems; benzo(g.h.i)perylene 66.4 µg/kg in seeds, 67.8 µg/kg in leaves and 28.2 µg/kg in stems). Deelaman et al. (2023) measured PAH concentrations in paddy grain samples from Thailand and Laos. The dominance of HMW PAHs was experienced: the five-ring benzo(k)fluoranthene was found in the highest concentration in samples collected in Thailand, and dibenzo(a.h)anthracene in samples from Laos.

The effect of road traffic on PAH accumulation has been experimentally tested. Dan-Badjo et al. (2007) used ryegrass (*Lolium perenne*) in a pot study in the vicinity of a highway and found significant accumulation of high molecular weight PAHs*. Lactuca sativa* plants were placed in a pot experiment nearby a highway in an early study of Larsson and Sahlberg (1981) showing high accumulation of benzo(g.h.i)perylene (10.8 μg/kg).

Calculated BCF values (Table 2) also show the higher accumulation rate of 6-ring PAHs, reaching as much as 1321 in seeds, 987.88 in leaf and 981.82 in stem in case of indeno1.2.3CD-pyrene, and 1897 in seeds, 1937 in leaf and 805.71 in stem in case of benzo(g.h.i)perylene. A previous study (Teke et al. 2020) used *Lactuca sativa* L. as test plant, treated with diesel extract prepared in a similar way. *Lactuca* showed higher accumulation rate expressed as higher BCFs for lower molecular weight PAHs. Comparative studies between mustard and lettuce accumulation potential indicate, however, that mustard is more efficient in accumulation HMW PAHs than lettuce (unpublished data).

**Table 2:** Calculated BCF values of 3–6-ring PAHs for different plant matrices

	PAH	BCF seed	BCF leaf	BCF stem	Molecular weight [g/mol]
Three rings	Acenaphthylene	74.17	180.83	10.00	152.19
	Acenaphthene	138.46	30.77	13.46	154.21
	Fluorene	25.21	11.86	2.97	166.22
	Phenanthrene	89.06	87.05	40.63	178.23
	Anthracene	210.34	77.59	31.03	178.23
Four rings	Fluoranthene	389.19	347.3	159.46	202.25
	Pyrene	317.28	206.17	82.72	202.25
	Benzanthracene	593.75	496.88	37.19	228.29
	Chrysene	1 469	976.92	461.54	228.30
Five rings	Benzo(b)fluoranthene	798.44	575.00	232.81	252.31
	Benzo(k)fluoranthene	494.44	541.67	216.67	252.31
	Benzo(e)pyrene	92.73	130.91	78.18	252.32
	Benzo(a)pyrene	358.14	283.72	148.84	252.32
	Dibenzo(a.h)anthracene	771.11	1 691	217.78	276.33
Six rings	Indeno1.2.3CD-Pyrene	1 321	987.88	981.82	276.30
	Benzo(g.h.i)perylene	1 897	1 937	805.71	278.35

As concluding remarks, it can be stated that high accumulation of PAHs in mustard seeds has been experimentally proven. The design of the study, as it involves spraying the above-ground parts of the test plants, excludes PAHs transport from the soil, however, it cannot distinguish clearly whether seeds directly absorb PAHs from the extract used for spraying or from the leaf-stem compartments. In general, atmospheric PAH contamination shows a clear seasonal pattern, having two important sources in colder seasons (biomass burning for household heating and traffic), whereas traffic is the main contributor during the vegetation period. As such, results emphasize the potential environmental impact of traffic-related PAH emissions on the quality of mustard cultivated in near-road agricultural fields.
